# SORL1 is a receptor for tau that promotes tau seeding

**DOI:** 10.1016/j.jbc.2024.107313

**Published:** 2024-04-23

**Authors:** Joanna M. Cooper, Aurelien Lathuiliere, Enming J. Su, Yuyu Song, Daniel Torrente, Youhwa Jo, Nicholas Weinrich, Jennifer Diaz Sales, Mary Migliorini, Thomas H. Sisson, Daniel A. Lawrence, Bradley T. Hyman, Dudley K. Strickland

**Affiliations:** 1 The Center for Vascular and Inflammatory Diseases, University of Maryland School of Medicine, Baltimore, Maryland, USA; 2 Alzheimer Research Unit, Department of Neurology, Massachusetts General Hospital and Harvard Medical School, Charlestown, Massachusetts, USA; 3 Department of Rehabilitation and Geriatrics, Memory Center, Geneva University Hospital and University of Geneva, Geneva, Switzerland; 4 Department of Internal Medicine, University of Michigan School of Medicine, Ann Arbor, Michigan, USA; 5 Patricia and John Rosenwald Laboratory of Neurobiology and Genetics, The Rockefeller University, New York, New York, USA; 6 Department of Physiology, University of Maryland School of Medicine, Baltimore, Maryland, USA; 7 Department of Surgery, University of Maryland School of Medicine, Baltimore, Maryland, USA

**Keywords:** tau protein (tau), tauopathy, neurodegenerative disease, tau seeding, lipoprotein receptor-related protein (LRP), apolipoprotein E (ApoE), SORL1

## Abstract

Sortilin-related receptor 1 (SORL1) is an intracellular sorting receptor genetically implicated in Alzheimer’s disease (AD) that impacts amyloid precursor protein trafficking. The objective of these studies was to test the hypothesis that SORL1 binds tau, modulates its cellular trafficking and impacts the aggregation of cytoplasmic tau induced by pathological forms of tau. Using surface plasmon resonance measurements, we observed high-affinity binding of tau to SORL1 and the vacuolar protein sorting 10 domain of SORL1. Interestingly, unlike LDL receptor-related protein 1, SORL1 binds tau at both pH 7.4 and pH 5.5, revealing its ability to bind tau at endosomal pH. Immunofluorescence studies confirmed that exogenously added tau colocalized with SORL1 in H4 neuroglioma cells, while overexpression of SORL1 in LDL receptor-related protein 1-deficient Chinese hamster ovary (CHO) cells resulted in a marked increase in the internalization of tau, indicating that SORL1 can bind and mediate the internalization of monomeric forms of tau. We further demonstrated that SORL1 mediates tau seeding when tau RD P301S FRET biosensor cells expressing SORL1 were incubated with high molecular weight forms of tau isolated from the brains of patients with AD. Seeding in H4 neuroglioma cells is significantly reduced when SORL1 is knocked down with siRNA. Finally, we demonstrate that the N1358S mutant of SORL1 significantly increases tau seeding when compared to WT SORL1, identifying for the first time a potential mechanism that connects this specific SORL1 mutation to Alzheimer’s disease. Together, these studies identify SORL1 as a receptor that contributes to trafficking and seeding of pathogenic tau.

The pathological hallmarks of Alzheimer’s disease (AD) include the presence of extracellular amyloid plaques composed of aggregated forms of the Aβ peptide and intracellular neurofibrillary tangles composed of hyperphosphorylated, misfolded, and aggregated forms of microtubule-associated protein tau within the cytoplasm of certain neurons. The aberrant assembly of tau into insoluble aggregates is associated with a diverse group of neurodegenerative diseases that include AD. In the case of AD, the neuronal transfer of pathological forms of tau has been proposed as a mechanism of AD progression, as the accumulation of misfolded tau aggregates initiates in the entorhinal cortex and spreads across connected neural pathways (1, 2, 3, 4, 5, 6, 7). This spreading is thought to be mediated by the cell-to-cell transfer of proteopathic seed-competent tau. How these forms of tau are taken up and delivered to the cytoplasm of the recipient cells remain the central question in understanding this process, but likely involves cellular receptors that internalize tau (8, 9). Recent studies have demonstrated that the low-density lipoprotein (LDL) receptor-related protein 1 (LRP1) is a key receptor that can mediate tau uptake across neural systems (10, 11). Cell-based studies revealed that upon binding of monomeric tau, LRP1-mediated uptake leads to efficient degradation of this protein (11). This study also noted that LRP1-expressing cells, but not cells deficient in LRP1, promoted cytosolic tau aggregation when incubated with modified tau derived from brain lysates of human AD brain tissue extracts (11) when using HEK293T FRET biosensor cells that stably express the P301S FRET biosensor, a commonly used to assay to detect tau seeding activity (12).

We previously observed residual uptake of ^125^I-labeled tau in cells genetically deficient in LRP1 (11), revealing the existence of additional receptors capable of mediating tau uptake. We hypothesized that sortilin-related receptor 1 (SORL1, SORLA, or LR11, gene name *SORL1*), a protein structurally related to LRP1 and itself a protein that is genetically linked to AD (13, 14, 15), might participate in tau trafficking. SORL1 is a 250 kDa type-1 transmembrane sorting receptor that shuttles between the *trans*-Golgi network (TGN), cell surface, and endosomes (16, 17), and is expressed in neurons and microglia along with several other cell types (18). SORL1 is primarily localized to intracellular compartments, with ∼10% of SORL1 expressed on the cell surface (19). SORL1 is a member of a family of vacuolar protein sorting 10 (VPS10) domain containing receptors and contains multiple functional domains, some of which confer structural similarity to LRP1. In addition to the VPS10 domain, SORL1 also contains a β-propeller domain, an epidermal growth factor-type domain, a cluster of LDL ligand binding repeats similar to those found in LRP1, fibronectin type III domains, a transmembrane domain and an intracellular domain containing recognition sites for cytosolic adaptor proteins (16, 20).

SORL1 is associated with both early and late onset forms of AD and is a genetic risk factor for late onset, sporadic AD (15, 21, 22, 23, 24). Substantial evidence has revealed that loss of SORL1 impacts amyloid precursor protein (APP) trafficking and regulates the production of amyloid β (Aβ) levels in the brain (25), but potential interactions between SORL1 and tau remain unstudied. Interestingly, SORL1 haploinsufficiency in Gottingen minipigs resulted in elevated levels of Aβ as well as tau in the cerebral spinal fluid (26). Genome-wide association studies and whole exome sequencing studies have identified common and rare single nucleotide polymorphisms in *SORL1* that are associated with early onset familial AD (14, 22, 27, 28, 29). Among these are the N1358S mutation in the *SORL1* gene which was identified in an exome sequencing study of patients with early onset AD. Until now the functional consequences of this mutation have not been identified (14).

To test our hypothesis that SORL1 is involved in tau trafficking, we utilized surface plasmon resonance (SPR) measurements that confirmed high-affinity binding of tau to SORL1. We further found that the expression of SORL1 in LRP1-deficient Chinese Hamster Ovary (CHO) and H4 neuroglioma cells leads to enhanced tau internalization, and that fluorescently labeled tau colocalizes with endogenously expressed SORL1 in H4 cells. Finally, we discovered that SORL1 promotes cytosolic tau seeding induced by pathogenic forms of tau, which was significantly increased by the N1358S mutant of SORL1. Together, these studies identify SORL1 as a receptor that contributes to trafficking and seeding of pathogenic tau.

## Results

### Tau binds to SORL1 with high affinity

**Figure 1:**
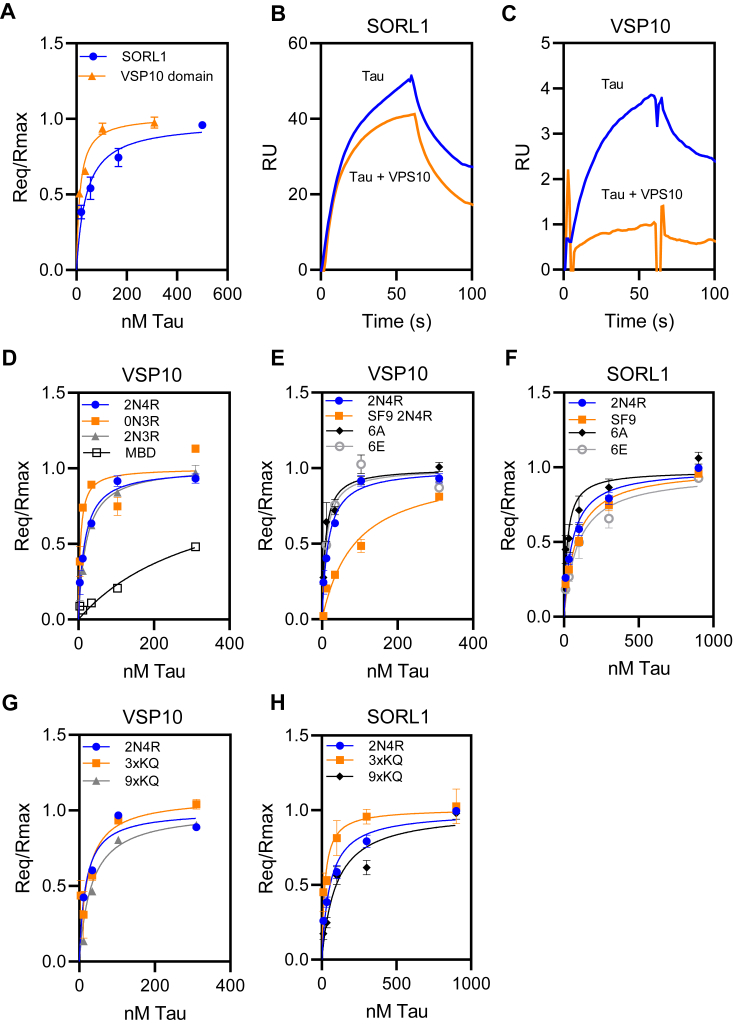
**Recombinant monomeric tau binds to SORL1 and the VPS10 domain of SORL1.***A*, equilibrium analysis of the binding of increasing concentrations of recombinant 2N4R tau to full-length SORL1 (*blue circles*) and the SORL1 VPS10 domain (*orange triangles*) coupled to a Biacore CM5 sensor chip. *b* and *c*, recombinant SORL1 (*B*) or VPS10 (*C*) was immobilized on the chip and binding of 25 nM tau ± 500 nM VPS10 was assessed. Shown are representative images. *D*, binding of tau isoforms 2N4R, 0N3R, 2N3R, and tau MBD to SORL1 VPS10 domain assessed by SPR equilibrium analysis. *E* and *F*, the binding of tau produced by Sf9 cells along with two mutant forms of tau to SORL1 VPS10 domain (*E*) or SORL1 (*F*) was measured by SPR; 6A (T181, S199, S202, S396, S400, and S404 are all converted to alanine) and 6E, in which all these residues are converted to glutamic acid. *G* and *H*, binding of mutant forms of tau to SORL1 VPS10 domain (*G*) or SORL1 (*H*): 3XKQ in which lysine residues 311, 317, and 321 were converted to glutamine residues and 9XKQ tau in which lysine residues 311, 217, 321, 340, 343, 347, 353, 369, and 375 are all converted to glutamine residues. The data were normalized to Rmax to correct for slight differences in coupling to the SPR surfaces. Each experiment was repeated at least three times. Shown are mean ± SEM. MBD, microtubule-binding domain; SORL1, sortilin-related receptor 1; SPR, surface plasmon resonance; VPS10, vacuolar protein sorting 10.

To determine if SORL1 can directly bind tau, we used SPR experiments. In these experiments, recombinant human SORL1 (residues 82–2135) was coupled to an SPR chip and increasing concentrations of 2N4R tau were flowed over the surface. The data revealed that recombinant 2N4R tau bound tightly to SORL1, with a K_D_ value of 59 ± 18 nM (Fig. 1*A*, and Table 1), close to the K_D_ of 60 ± 8 nM reported for the interaction of LRP1 with tau (11). We found that this binding event did not fit a simple 1:1 binding model, and thus we used equilibrium binding analysis to determine the K_D_.

**Table 1:** Kinetic and equilibrium binding constants for the interaction of various forms of tau with the VPS10 domain of SORL1 and full length SORL1^a^

Tau	Mutants	k_a_ (M^−1^s^−1^) VPS10 domain	k_d_ (s^−1^) VPS10 domain	K_D_ (nM) VPS10 domain^b^	K_D eq_ (nM) VPS10 domain^c^	K_D eq_ (nM) Full length SORL1^c^
2N4R	WT	5.0 ± 7.0 × 10^7^	4.1 ± 5.5 × 10^−1^	15 ± 9	17 ± 1	59 ± 18
0N3R	Tau lacking N1 and N2	1.3 ± 0.3 × 10^5^	1.3 ± 0.4 × 10^−2^	10 ± 1	5 ± 1	nd
2N3R	Tau lacking R2	1.9 ± 0.4 × 10^6^	1.1 ± 0.6 × 10^−2^	18 ± 3	11 ± 2	nd
MBD	L243-E372	2.8 ± 0.5 × 10^5^	2.4 ± 0.6 × 10^−3^	93 ± 40	341 ± 14	nd
2N4R SF9	Tau produced in SF9 cells	8.5 ± 2.5 × 10^4^	6.1 ± 2.9 × 10^−3^	78 ± 13	95 ± 28	104 ± 36
2N4R 6A	T181/S199/S202/S396/S400/S404 to A	1.1 ± 0.5 × 10^6^	1.1 ± 0.7 × 10^−2^	10 ± 1	9 ± 2	32 ± 26
2N4R 6E	T181/S199/S202/S396/S400/S404 to E	8.3 ± 1.8 × 10^5^	1.5 ± 0.5 × 10^−2^	18 ± 3	11 ± 2	116 ± 42
2N4R 3×KQ	K311/317/321 to Q	1.5 ± 0.6 × 10^6^	2.0 ± 0.6 × 10^−2^	14 ± 2	17 ± 12	21 ± 9
2N4R 9×KQ	K311/317/321/340/343/347/353/369/375 to Q	7.0 ± 2.0 × 10^5^	1.5 ± 0.3 × 10^−2^	22 ± 6	32 ± 2	100 ± 23

Abbreviations: A, alanine; E, glutamic acid; nd, not determined; Q, glutamine.

a Average of at least three-independent experiments ± STD.

b The equilibrium dissociation constant K_D_ was calculated from the kinetic parameters for a 1:1 fit.

c K_D_ eq was calculated from SPR equilibrium measurements, in which Req was determine by fitting the association data to a pseudo-first order process.

The VPS10 domain of SORL1 is a ligand binding site that directly binds to the amyloid-β peptide (30). This domain forms a ten-bladed β-propeller fold that preferentially recognizes β-sheet forming peptides (31) and is structurally distinct from the complement binding repeat clusters that form LRP1 ligand binding sites. Therefore, we conducted experiments to determine if tau can bind to SORL1’s VPS10 domain, and if various AD-associated mutations and posttranslational modifications impact this binding. To accomplish this, we immobilized the recombinant VPS10 domain of SORL1 (residues 82–753) on an SPR chip and flowed increasing concentrations of tau over the surface. Kinetic analysis of tau binding VPS10 domain follows a simple 1:1 binding model. The results reveal that the VPS10 domain binds tau with a K_D_ value even tighter than that of the full-length molecule (K_D_ = 17 ± 1 nM) (Fig. 1*A*, and Table 1).

We next conducted experiments to determine if the VPS10 domain can compete for the binding of tau to SORL1. Recombinant SORL1 protein was immobilized on an SPR sensor chip, and the binding of tau was assessed in the presence or absence of saturating concentrations of the VPS10 domain. The results of this experiment reveal that the VPS10 domain only partially competes for the binding of tau to SORL1 (Fig. 1*B*), confirming that tau interacts with SORL1 at multiple sites. As a control the same binding assay was repeated on an SPR sensor chip coated with VPS10 peptide, and the results confirm that saturating concentrations of VPS10 in solution block the binding of tau to VPS10 on the chip (Fig. 1*C*).

Tau consists of an N-terminal “projection” domain that contains alternatively spliced regions denoted as “N1” and “N2” regions (32), along with a C-terminal microtubule-binding domain (MBD) composed of four highly conserved repeat regions, “R1”–“R4”. The MBD is the site of interaction for tau with microtubules, and also a primary site for the interaction of tau with LRP1 (11). To identify domains in tau responsible for its interaction with SORL1, we investigated the binding of 0N3R (lacking the two N-terminal inserts), 2N3R (lacking the second R domain and which has reduced affinity for LRP1 (11), and the MBD (R1-R4, leu243-glu372) to the SORL1 VPS10 domain, and obtained K_D_ values of 5 ± 1, 11 ± 2, and 341 ± 14 nM, respectively (Fig. 1*D*). These results reveal that, distinct from LRP1, the major tau binding site for SORL1 is not *via* tau’s MBD and that tau binding to SORL1 is not impacted by removal of the second R domain.

Tau is a highly posttranslationally modified protein, containing multiple serine, threonine, and tyrosine phosphorylation sites that have been extensively studied and that are detected in tau aggregates in AD and other tauopathies. In our previous study, we found that hyperphosphorylation of tau significantly reduces its affinity for LRP1 (11). To investigate if phosphorylation of tau impacts tau binding to SORL1, we examined the binding of recombinant tau produced in SF9 insect cells, which produce well-characterized hyperphosphorylated forms of tau (33, 34). We found SF9 tau binds SORL1 VPS10 with a K_D_ of 95 ± 28 nM (Fig. 1*E*) and to SORL1 with a K_D_ of 104 ± 36 nM (Fig. 1*F*). We also examined the binding of two mutant forms of tau to SORL1 VPS10 domain and to SORL1: mutant 6A, in which T181, S199, S202, S396, S400, and S404 are all converted to alanine, and mutant 6E, in which all these residues are converted to the phosphomimetic glutamic acid. These specific residues have been found to be phosphorylated to a greater extent in AD brains (35), and we previously found the 6E mutant binds LRP1 with weaker affinity, while 6A binds LRP1 similar to WT tau (11). Our results reveal that the affinity of either mutant for the SORL1 VPS10 domain is like that of the 2NR4 tau (6A mutant; K_D_ = 9 ± 2 nM; 6E mutant K_D_ = 11 ± 2 nM) (Fig. 1*E*), but slightly weaker to full-length SORL1 (6A mutant; K_D_ = 32 ± 26 nM; 6E mutant K_D_ = 116 ± 42 nM) (Fig. 1*F*).

Lysine residues are important for the binding of tau to LRP1 (10, 11). To determine if lysine residues are involved in the interaction of tau with SORL1, we examined the binding of mutated forms of tau in which lysine residues 311, 317, and 321 were all converted to glutamine residues (3XKQ) and a form of tau in which lysine residues 311, 217, 321, 340, 343, 347, 353, 369, and 375 are all converted to glutamine residues (9XKQ). Both of these mutant tau molecules displayed weaker binding to LRP1 (11). In contrast, the binding of these mutated tau proteins to the SORL1 VPS10 domain was only slightly impaired (K_D_ 3XKQ mutant = 17 ± 12 nM; K_D_ 9XKQ mutant = 32 ± 2 nM) (Fig. 1*G*) but the 9XKQ mutant bound slightly weaker to full-length SORL1 (K_D_ 3XKQ mutant = 21 ± 9 nM; K_D_ 9XKQ mutant = 100 ± 23 nM) (Fig. 1*H*). Kinetic parameters and equilibrium K_D_ values the fits of various forms of tau binding to the SORL1 VPS10 domain and SORL1 are summarized in Table 1.

### SORL1 can mediate the endocytosis of tau

**Figure 2:**
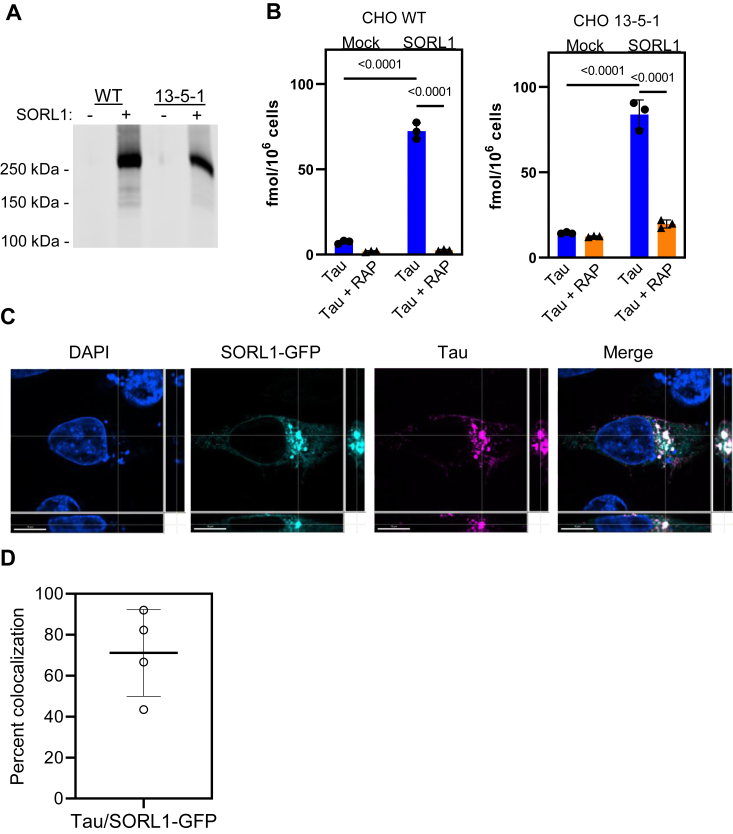
**SORL1 mediates the internalization of tau and colocalizes with internalized tau.** WT CHO and LRP1-deficient CHO 13-5-1 cells were transfected with SORL1 plasmid or empty vector (Mock). *A*, transfection efficiency was validated *via* immunoblot analysis. *B*, transfected cells were incubated with 20 nM ^125^I-labeled tau ± 1 μM RAP for 2 h, and the amount of tau internalized by the cells was quantified. (Mean ± SEM, two-way ANOVA followed by Tukey multiple comparisons test). *C*, H4 cells were transfected with SORL1-GFP plasmid, then incubated with 40 nM tau labeled with Alexa Flour 594 for 2 h. Cells were fixed and imaged on a Nikon spinning disk confocal microscope at 60×. Cells expressing SORL1 (*teal*) internalize more tau (*magenta*) than neighboring cells not expressing SORL1-GFP (nuclei marked by DAPI staining, *blue*). Tau and SORL1 colocalization is represented in *white* in the merged image. The scale bar represents 10 μm. *D*, single cell analysis of the percent of tau that colocalizes with SORL1-GFP using Imaris Bitplane Software. Shown is mean ± SD, n = 4. CHO, Chinese hamster ovary; DAPI, 4′,6-diamidino-2-phenylindole; LDL receptor-related protein 1; RAP, receptor associated protein; LRP1; SORL1, sortilin-related receptor 1.

When cells are transfected with SORL1, this receptor can mediate the endocytosis of ligands (36). To test the hypothesis that cellular forms of SORL1 can bind tau and mediate its internalization, we initially chose to conduct experiments in LRP1-deficient CHO cells (CHO 13-5-1) (37) which do not express detectable levels of SORL1. These cells, along with WT CHO cells were transfected with a plasmid containing a construct that encoded SORL1 (Fig. 2*A*). Incubation of the cells with ^125^I-labeled tau for 2 h at 37 °C revealed that both CHO WT and CHO 13-5-1 cells expressing SORL1 demonstrated a dramatic increase in the amount of tau internalized when compared to cells incubated with transfection reagent only (“mock”) (Fig. 2*B*). The internalization of tau in SORL1-transfected cells was inhibited by the receptor associated protein (RAP), an inhibitor of LDL receptor family members which is known to bind tightly to SORL1 and antagonize certain ligands from interacting with this receptor (38).

To determine if tau colocalizes with SORL1 after internalization, we transfected H4 neuroglioma cells with a SORL1-GFP construct and then incubated the transfected cells with 40 nM Alexa Flour 594-labeled 2N4R tau. After 2 h of incubation, the cells were fixed and analyzed by confocal microscopy. The results show a marked degree of colocalization of tau with SORL1 in the transfected cells (Fig. 2*C*). Single-cell analysis of the degree of colocalization using Imaris Bitplane (https://imaris.oxinst.com/) software revealed 71.11 ± 21% colocalization of intracellular tau with SORL1 (Fig. 2*D*).

### SORL1 knockdown in H4 cells does not affect the uptake of ^125^I-tau

**Figure 3:**
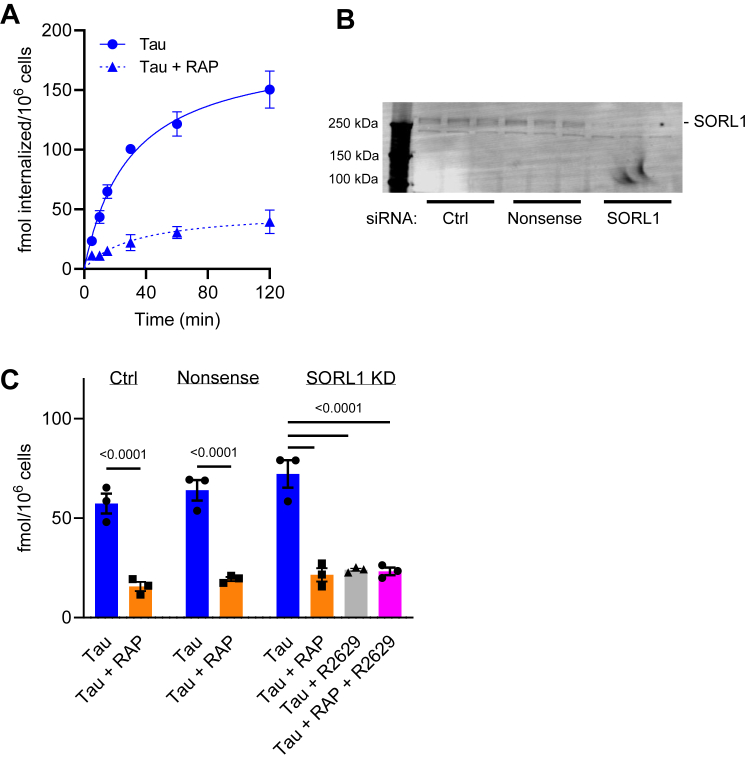
**SORL1 knockdown does not impact tau uptake in H4 cells.***A*, time course for ^125^I-tau internalization in H4 cells. Cells were incubated with 20 nM ^125^I-labeled tau ± 1 μM RAP for designated times and internalized tau measured. *B* and *C*, H4 cells were treated with vehicle control, nonsense siRNA, or siRNA targeting SORL1, then incubated with 20 nM ^125^I-labeled tau ± 1 μM RAP or 300 μg/ml R2629 for 2 h. *B*, SORL1 knockdown confirmed by immunoblot analysis. *C*, internalized tau was quantified. Shown are mean ± SEM; 2-way ANOVA, Tukey’s multiple comparison test, n = 3. RAP, receptor associated protein; SORL1, sortilin-related receptor 1.

Having demonstrated the ability of SORL1, when overexpressed in cells, to bind and mediate tau internalization we next focused studies on determining if endogenously expressed SORL1 associates with tau in a central nervous system relevant model. For these experiments, we used the neuroglioma H4 cell line which expresses both LRP1 and SORL1. Our initial experiments measured the time course of ^125^I-labeled tau internalization in H4 cells in the presence or absence of RAP, and the data reveal that steady-state levels of tau are reached around 2 h of incubation at 37 °C (Fig. 3*A*). To gain insight into the role of SORL1 in tau processing, we used siRNA to knockdown SORL1 in H4 neuroglioma cells and then measured the impact on the receptor-mediated internalization of ^125^I-labeled tau. Knockdown efficiency was confirmed *via* immunoblotting (Fig. 3*B*). To assess the contribution of LRP1 to this process, we inhibited LRP1 function with an anti-LRP1 immunoglobulin G (IgG) (R2629) (Fig. 3*C*). The results reveal that although we observed effective knockdown of SORL1 (Fig. 3*B*), we noted little impact on ^125^I-tau internalization in the cells lacking SORL1 (Fig. 3*C*). Virtually all of the internalized tau was blocked with anti LRP1 IgG. These results reveal that in H4 cells, tau internalization is primarily mediated *via* an LRP1-mediated process, and are consistent with the fact that SORL1 is primarily localized to intracellular compartments (19).

### Internalized tau colocalizes with endogenous SORL1 in H4 cells

**Figure 4:**
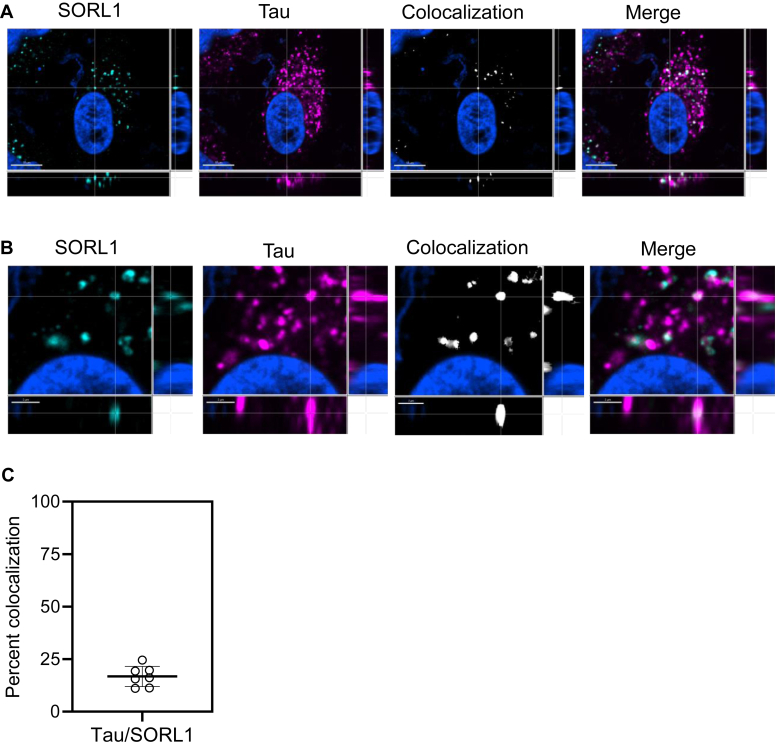
**Tau colocalizes with endogenous SORL1 in H4 cells.***A*, H4 cells were incubated with 40 nM tau labeled with Alexa Flour 594 for 2 h, then fixed and immunostained with anti-SORL1 antibody to label endogenous SORL1. The scale bar represents 10 μm. (*B*) Inset from panel (*A*) zoomed in to 200%, scale bar is 2 μm. All cells were fixed and imaged on Nikon spinning disk confocal microscope at 60×. Imaris Bitplane software was used to identify colocalization (*white*) between SORL1 (*teal*) and tau (magenta). Nuclei marked by DAPI staining, *blue*. *C*, single-cell analysis of the percent of tau that colocalizes with SORL1. Shown is mean ± SD, n = 7. DAPI, 4′,6-diamidino-2-phenylindole; SORL1, sortilin-related receptor 1.

To determine if endogenous SORL1 colocalizes with tau following its internalization in H4 cells, we treated these cells with 40 nM Alexa Flour 594-labeled 2N4R tau for 2 h. Following incubation, the cells were fixed and stained for SORL1 using the D8D4G Rabbit anti-SORL1 antibody (Fig. 4, *A* and *B*). Analysis of the extent of colocalization of internalized tau with endogenous SORL1 was conducted using Imaris Bitplane (https://imaris.oxinst.com/) software reveals 17 ± 5% colocalization (Fig. 4*C*).

### SORL1 binds tau at endosomal pH

**Figure 5:**
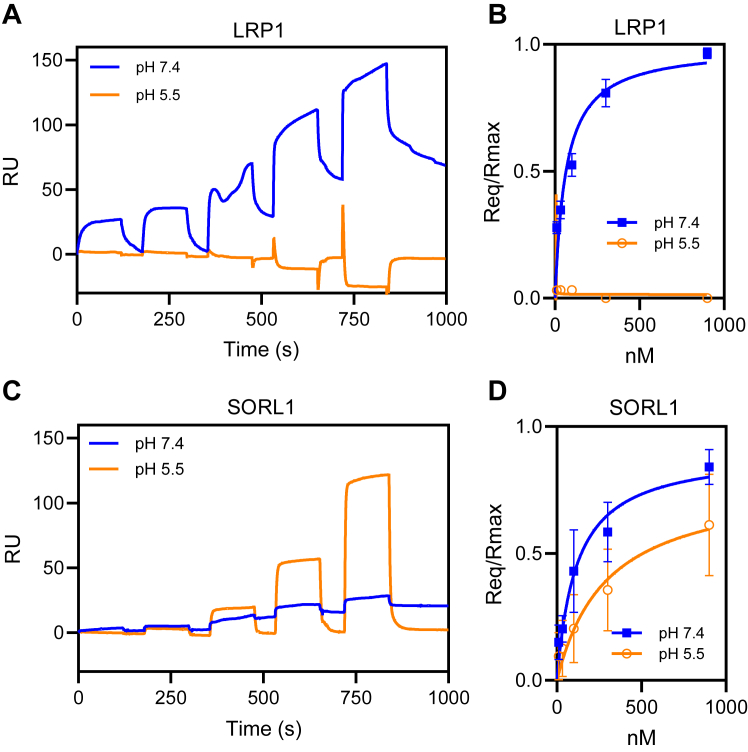
**Tau binds SORL1 at pH 7.4 as well as pH 5.5.** SPR analysis of tau binding LRP1 or SORL1 at pH 7.4 or pH 5.5. Increasing concentrations of tau (11, 33, 100, 300, and 900 nM were titrated on CM5 sensor chips coated with LRP1 (*A* and *B*) or SORL1 (*C* and *D*). The experiment was performed at pH 7.4 (*blue lines*) or pH 5.5 (*orange lines*). *B* and *C*, equilibrium fits of the data in panels a and c. Shown are mean ± SEM, n = 3. LRP1, LDL receptor-related protein 1; SORL1, sortilin-related receptor 1; SPR, surface plasmon resonance.

Since the internalization of tau in H4 cells is primarily mediated *via* an LRP1-dependent process and we note colocalization of internalized tau with endogenous SORL1 in these cells, this raised the possibility that tau internalized *via* an LRP1-mediated process may be transferred to SORL1 within endosomal compartments. To investigate this further, we examined the pH-dependency of binding of tau to both LRP1 and to SORL1. In these experiments, LRP1 and SORL1 were immobilized on SPR chips and incubated with increasing concentrations of tau at pH 7.4 and pH 5.5. The results revealed that, as previously shown, while LRP1 binds tau avidly at pH 7.4, this receptor is not capable of binding tau at pH 5.5. In striking contrast, SORL1 binds tightly to tau at both pH 7.4 and pH 5.5 (Fig. 5).

### SORL1 supports tau proteopathic seeding in the cytoplasm

**Figure 6:**
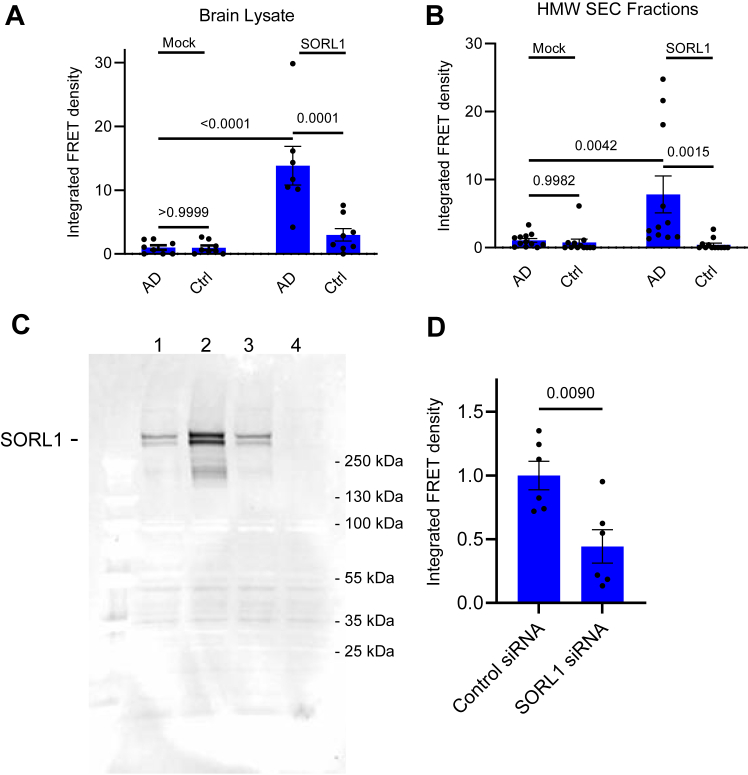
**SORL1 transfection reconstitutes pathogenic internalization and seeding in HEK293T reporter cells.** HEK293T Tau RD P301S FRET Biosensor cells were transfected with *SORL1*, then incubated with (*A*) human brain homogenate from an Alzheimer’s patient (AD) or age-match healthy control (Ctrl) fraction or (*B*) HMW SEC fractions from AD patient brain (AD) or healthy control (Ctrl) and tau seeding was quantified. *C*, siRNA was used to knockdown SORL1 in H4 cells and was confirmed by immunoblot analysis, SORL1 band marked. Lane 1, nontransfected, lane 2, transfected with pcDNA SORL1, lane 3, control siRNA, and lane 4, SORL1 siRNA. *D*, H4 cells stably expressing the FRET reporter system with SORL1 knockdown were incubated with 300 ng/well AD brain derived HMW tau seeding material and tau seeding was quantified. (Mean ± SEM; 2-way ANOVA, Tukey’s multiple comparison test). SEC, size exclusion chromatography; HMW, high molecular weight; SORL1, sortilin-related receptor 1.

A key to understanding SORL1’s role in the propagation of tau pathology is the question of whether SORL1 expression can promote tau seeding. To determine this we utilized HEK293T FRET biosensor cells that stably express the P301S FRET biosensor and are commonly used to assay tau seeding activity (12). We conducted experiments in which these cells were transfected with SORL1 and incubated with brain lysate from Braak VI AD patients (Fig. 6*A*) or with high molecular weight (HMW) fractions obtained following size exclusion chromatography (SEC) isolated from brains of AD patients or healthy age-matched control (Fig. 6*B*) (8, 9). Western blot characterization of brain lysate and HMW or low molecular weight SEC fractions are included in Fig. S1. The results reveal that expression of SORL1 in transfected HEK293T biosensor cells results in a significant increase in tau seeding induced by brain lysates or HMW SEC fractions from AD brains.

To determine if SORL1 impacts tau seeding when endogenously expressed, we used siRNA to knockdown SORL1 in H4 neuroglioma cells that stably express the P301S FRET biosensor (Fig. 6*C*). We found that deletion of SORL1 in H4 cells significantly reduced tau seeding induced by AD homogenates when compared with cells expressing SORL1 (Fig. 6*D*). These experiments reveal that SORL1 supports seeding of pathological forms of tau, presumably *via* endolysosomal escape.

### SORL1 harboring N1358S mutation exhibits increased tau seeding in HEK293T FRET reporter cells

**Figure 7:**
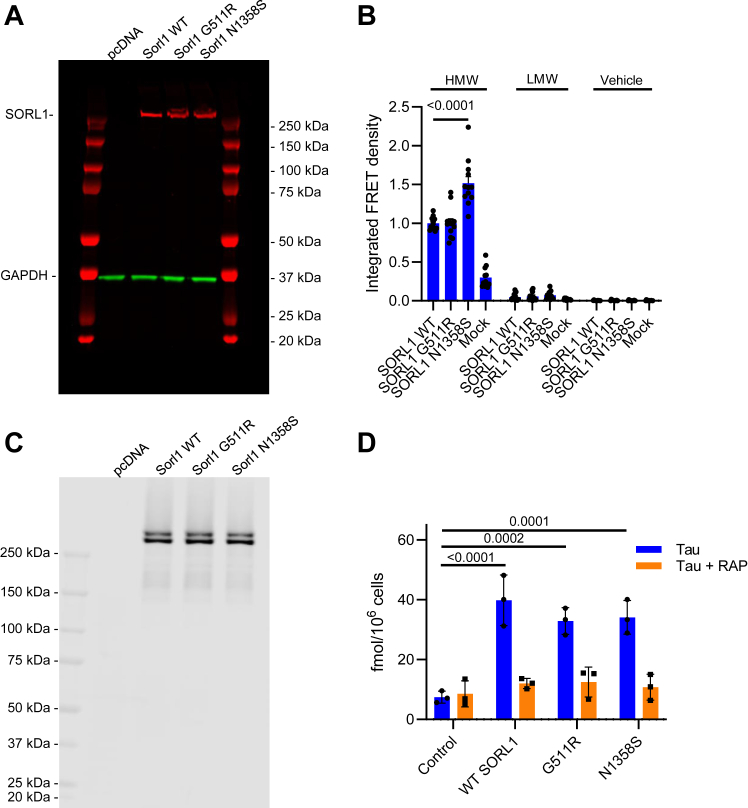
**SORL1 harboring the N1358S mutation exhibits increased seeding capacity in HEK293T cells.** HEK293T Tau RD P301S FRET Biosensor cells were transfected with plasmid containing WT SORL1 or SORL1 harboring the G511R or N1358S mutations. *A*, expression level of different variants was measured by Western blot (GAPDH was used as a loading control). These cells were incubated with (*B*) HMW or LMW SEC fractions from human AD patient brain or vehicle control and tau seeding was quantified. In a separate experiment, transfected cells expressing equal levels of the SORL1 variants (*C*) were incubated with (*D*) 20 nM ^125^I-labeled tau in the presence or absence of 1 μM RAP for 2 h and then internalized tau was quantified. (Mean ± SEM; 2-way ANOVA, Tukey’s multiple comparison test). AD, Alzheimer’s disease; HMW, high molecular weight; LMW, low molecular weight; RAP, receptor associated protein; SORL1, sortilin-related receptor 1.

We next investigated the ability of two SORL1 mutations, G511R and N1358S to mediate tau internalization and promote tau seeding. These mutant forms of SORL1 were identified by exome sequencing in patients with early onset AD. G511R is located in the VPS10 domain and impairs the ability of SORL1 to bind and facilitate lysosomal catabolism of Aβ (14, 30), while the N1358S mutation is located in the LDL ligand binding complement-type repeat 7 of SORL1, and it is unknown how this mutation affects receptor function (14). To investigate the seeding capacity of these mutant forms of tau, HEK293T FRET reporter cells were transfected with plasmids containing WT SORL1 or SORL1 harboring the G511R or N1358S mutations (Fig. 7*A*), and then incubated with HMW (highly phosphorylated soluble species that support seeding) or low molecular weight (minimally phosphorylated tau species that do not support tau seeding) (8) SEC fractions from AD patient brain or vehicle control. HEK293T biosensor cells transfected with N1358S SORL1 showed significant increases in tau seeding over cells transfected with WT SORL1 when incubated with HMW SEC fractions from AD brains (Fig. 7*B*). In contrast, there were no differences in tau seeding with expression of a second SORL1 mutant (G511R) (Fig. 7*B*). To investigate if these changes in tau seeding were associated with increased tau internalization, HEK293T FRET reporter cells transfected with WT, G511R, or N1358S SORL1 (Fig. 7*C*) were incubated with 20 nM ^125^I-labeled tau, and then internalized tau was quantified. No differences in tau uptake were observed for either mutant (Fig. 7*D*). Combined, these results suggest that the N1358S mutation leads to increased tau seeding, while not impacting tau internalization.

## Discussion

SORL1 is an endocytic and intracellular sorting receptor that recognizes numerous ligands, regulates APP trafficking, and is genetically associated with AD. Here, we investigated the hypothesis that SORL1 also functions as a receptor that modulates tau trafficking in cells and can promote tau seeding. Through *in vitro* SPR binding assays and cell-based internalization and seeding assays, this study identifies SORL1 as a novel receptor for tau that regulates tau seeding and raises the possibility that SORL1 plays an important role in modulating the trafficking of tau.

Several lines of evidence confirm a strong interaction between tau and SORL1. First, using SPR technology, our data reveal that tau binds tightly to SORL1 *via* multiple sites that include its VPS10 domain. Second, we demonstrate that expression of SORL1 in cells significantly increases tau endocytosis and immunofluorescence studies confirm that fluorescently labeled tau colocalizes with SORL1-GFP when incubated with transfected H4 cells. Third, we demonstrate that in H4 cells which express both LRP1 and SORL1 most, if not all, of tau is internalized *via* an LRP1-mediated process. Despite this, after 2 h of incubation, a significant portion of tau (17%) colocalizes with endogenous SORL1. To examine potential mechanisms of how this may occur, we performed SPR studies at endosomal pH and discovered that while LRP1 is unable to bind tau at endosomal pH, SORL1 binds avidly to tau at acidic pH. These results raise the possibility of transfer of internalized tau from LRP1 to SORL1 within endosomal compartments. Additional studies examining the localization of LRP1, SORL1, and tau within various subcellular compartments will be required to determine if this is indeed the case.

**Figure 8:**
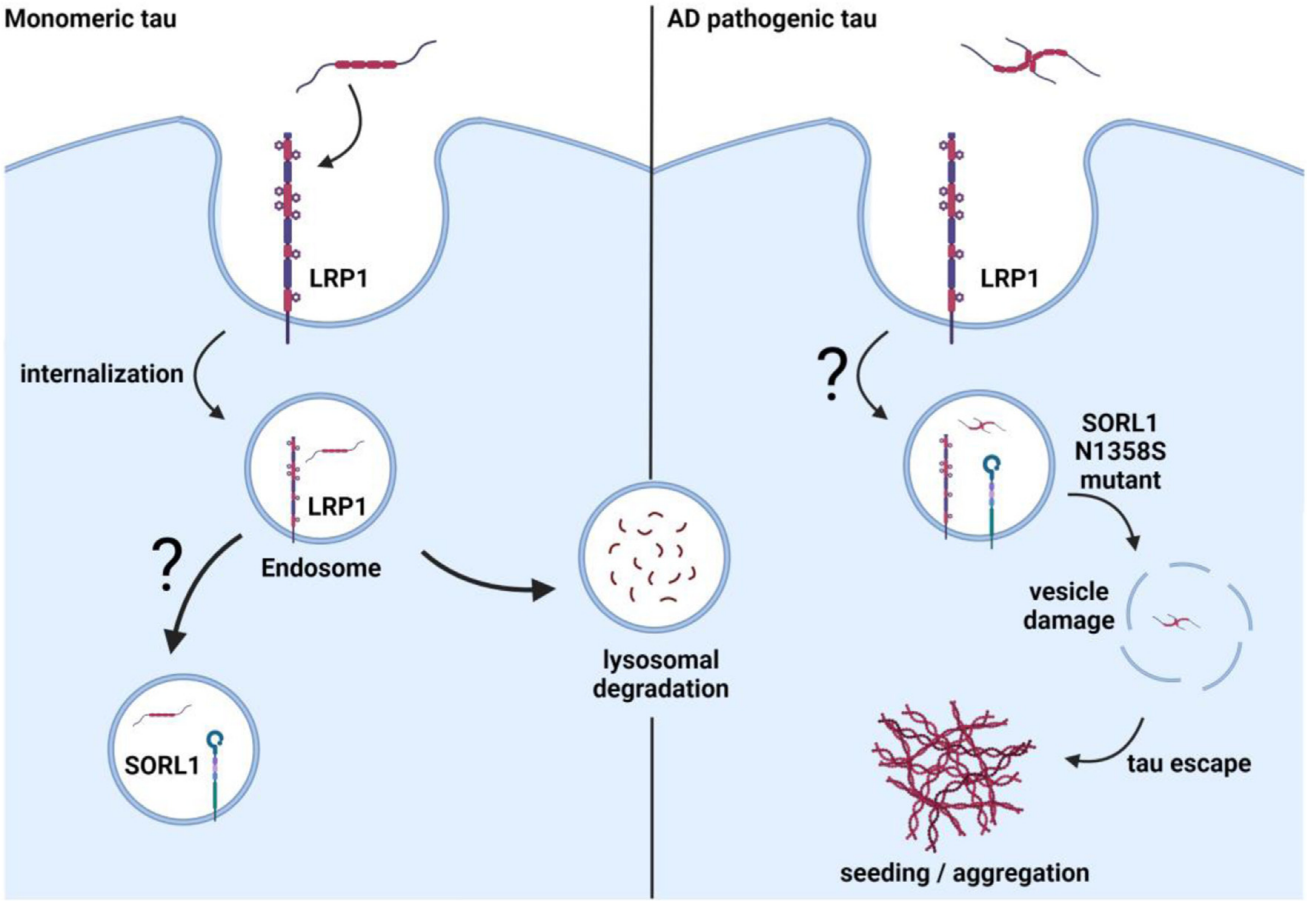
**Model of LRP1 and SORL1-mediated tau trafficking.** LRP1 mediates the internalization of monomeric forms of tau, resulting in rapid and efficient tau degradation. Internalized tau colocalizes with SORL1, in compartments that remain to be identified, and likely impacts tau trafficking through the endosomal pathways. Tau associates with SORL1 at endosomal pH of 5.5, but not LRP1, indicating a possible hand-off of tau from LRP1 to SORL1 as it traffics through endocytic pathways. LRP1 and SORL1 both mediate tau seeding in response to tau from human AD patient brains, and which could be through direct or indirect mechanisms. We postulate that AD pathogenic tau escapes the major degradative pathway to reach the extracellular space where it can induce cytoplasmic seeding. Furthermore, the AD-associated N1358S mutation in SORL1 increases the propensity of tau to seed aggregation, likely through impacting tau intracellular trafficking. Created with BioRender.com. AD, Alzheimer’s disease; LRP1, LDL receptor-related protein 1; SORL1, sortilin-related receptor 1.

The transfer of pathological forms of tau from neuron to neuron that leads to accumulation of misfolded intracellular tau aggregates is considered a potential mechanism by which AD progresses (1, 2, 3, 4, 5, 6, 7). A key step in that process is the ability of misfolded forms of tau to induce or “seed” the aggregation of endogenous cytoplasmic tau. This study reveals the importance of the SORL1/tau interaction in this process by demonstrating that SORL1 expression increases tau seeding, resulting in intracellular tau aggregation when cells are exposed to pathogenic forms of tau. The contribution of SORL1 to this process was further confirmed by our data showing that SORL1 knockdown also results in a decrease in cytoplasmic seeding induced by AD brain-derived HMW material containing seeding-competent tau. The mechanism underlying how SORL1 impacts tau seeding remains important future direction for this work. We hypothesize that LRP1 functions as a receptor that mediates tau endocytosis, directing tau to lysosomal pathways for rapid and efficient degradation (Fig. 8), as we noted in our previous study (11). One possible function of SORL1 may be to associate with internalized tau within endosomal compartments and regulate its sorting through endosomal compartments, mirroring its known function in regulating Aβ trafficking. In the case of hyperphosphorylated tau, this function could increase the propensity that tau will escape the endolysosomal pathway to allow enhanced seeding (Fig. 8). This hypothesis is consistent with our discovery that SORL1 is capable of binding tau at endosomal pH and with the proposed function of SORL1 which is to shuttle ligands between the TGN, endosomes and cell surface (39).

SORL1 is implicated in both early and late onset forms of AD and polymorphisms in *SORL1* are associated with early onset familial AD, and late-onset, sporadic AD (13, 14, 15). The N1358S mutation was identified once among AD cases in a study that included 15,808 AD cases and 16,097 control subjects from multiple European and American cohorts (40) and from exome sequencing of 14 autosomal dominant early-onset AD cases without any mutations on genes known to be causal for AD (*APP*, *PSEN1*, and *PSEN2*) (14). *In silico* predictions suggest that the N1358S variant is likely to have a pathogenic effect, but until now, the impact of the N1358S mutant on SORL1 function has not been identified. Our finding that SORL1 molecules harboring this mutation confers increased tau seeding capabilities to SORL1 provides a new molecular basis for understanding the genetic association of this mutant form of *SORL1* with AD. In contrast to the N1358S mutant, the G511R tau mutant had no effect on tau uptake or seeding. However, prior studies have suggested that cells expressing SORL1 molecules containing this mutation have enlarged lysosomes with impaired function and are deficient in Aβ binding, which is speculated to result in increased levels of Aβ resulting from reduced lysosomal degradation of Aβ (30, 41). As the list of AD-associated SORL1 SNPs grows, it will be interesting to determine if additional mutations have similar impacts on tau uptake and/or seeding.

How the N1358S mutation leads to increased seeding is a question of much interest. There could be multiple mechanisms explaining the increase in seeding that we observed. One possibility is that the N1358S mutation could disrupt tau binding to SORL1. Our binding data suggest that there are multiple binding sites on SORL1 for tau, one of them being the VPS10 domain, and it’s likely that a second sight is located within the LDL repeats since tau binding is inhibited by RAP which interacts with these repeats which are structurally related to the LDL repeats in LRP1. The N1358S mutation is in this LDL repeat, thus could impact tau binding. The mutation could also disrupt trafficking of the SORL1 receptor, but a study investigating the effects of various SORL1 mutations on protein processing determined that the N1358S variant did not affect SORL1 maturation in HEK293 cells (42). Our studies further support this finding as we observed no differences in the total expression levels of the N1358S mutation nor the amount of tau internalized when comparing N1358S to WT SORL1, suggesting that the differences we see in tau seeding are not related to different internalization efficiencies. A third possibility is that the N1358S mutation could impact tau trafficking out of the lysosome or retrograde to the TGN.

While our observations suggesting that the SORL1 gene may contribute to AD, they are seemingly in contrast to our understanding that SORL1 loss-of-function variants are considered causal AD. However, SORL1 has multiple important functions in biology. For example, SORL1 is an adaptor molecule for retromer trafficking complexes (43, 44), which are required for normal recycling of glutamate receptors and important process for synaptic plasticity and synaptic health (45). Additionally, the function of SORL1 is not limited to the brain, as SORL1 expressed in vascular smooth muscle cells regulates angiotensin II-mediated vascular pathology (46). An important remaining question is how internalized tau reaches the cytoplasm to seed intracellular tau aggregation. Abnormalities in the endo/lysosomal network are prevalent across neurodegenerative disorders (15, 47, 48, 49, 50), and interestingly, previous studies have found that the loss of SORL1 results in enlarged endosomes in human induced pluripotent stem cell-derived neurons and alters APP localization within the endosomal network (51). Certain mutations in SORL1 could disrupt tau trafficking, leading to aberrant accumulation of tau in endosomal compartments, and thus enable increased endosomal escape. While paradoxical, both loss-of-function and gain-of-toxic function mutations could exist in a receptor with multiple functions such as SORL1.

LRP1 and SORL1 have been found to interact with each other in perinuclear compartments of neurons (52), and it will be important to determine how these two receptors work together to regulate tau trafficking, as well as to understand how each receptor interacts with the various forms of tau. While tau binds both SORL1 and LRP1, our SPR experiments reveal key differences between the way each receptor interacts with tau. Collectively, our binding studies show that tau binds SORL1 with high affinity and reveals multiple binding sites on SORL1 for tau. Tau’s interaction with the SORL1 is different from its interaction with LRP1 in that while hyperphosphorylation has some impact on the affinity of tau for SORL1, this modification has a much greater impact on the affinity of tau for LRP1.

AD patient-derived tau is heterogeneous: tau exists in forms ranging from monomers to high molecular weight tau aggregates, each exhibiting different degrees of posttranslational modifications. Additional complexity is found interindividual variation in patterns of posttranslational modifications, which correlates with heterogeneity of disease progression (9). In induced plueropotent stem cells-derived neurons, deletion of SORL1 or various expressions of SORL1 mutants such as G511R, E270K, or Y141C does not result in changes in phosphorylated tau (41). Of particular importance will be to distinguish the differences between monomeric and pathogenic forms of tau and their abilities to interact with LRP1 and SORL1. Our current binding and cell-based uptake studies investigated recombinantly produced, monomeric forms of tau. In contrast, the seeding assay used a partially purified high molecular weight form of pathogenic form of tau derived from AD brains. These forms of tau are heavily posttranslationally modified. We have investigated how posttranslational modifications on tau impact its binding to both LRP1 and SORL1 using various phosphomimetic forms of tau. In general, we observed that increased phosphorylation or other posttranslational modifications reduce tau’s affinity for LRP1 but have a less substantial impact on SORL1 binding. It will be important in future experiments to determine if the contribution of LRP1 and SORL1 to increased seeding occurs *via* direct binding to pathogenic forms to these receptors, or *via* an indirect effect.

In conclusion, our studies identify a novel role for SORL1 as a receptor that mediates tau trafficking and demonstrate that SORL1 functions to promote tau seeding. Previous research into the role of SORL1 in AD has indicated that reduced brain levels of SORL1 increase the generation of Aβ by altering the transport of APP, and our finding that SORL1 also increases tau seeding highlights a paradox that lowering SORL1 levels might have both positive and negative effects in the progression of AD. The potential of this receptor to regulate trafficking of both APP and tau emphasizes the complex relationship between SORL1, its ligands and the role each plays in the progression of AD.

## Experimental procedures

### Cells

CHO K1 (WT CHO) and CHO 13-5-1 cells (LRP1-deficient) (37) were maintained in Dulbecco's modified Eagle medium/Ham's F12 with L-glutamine (DMEM/F12; Corning 10–090-CM) supplemented with 10% fetal bovine serum (FBS; Sigma-Aldrich F-4135). The Tau RD P301S FRET Biosensor embryonic kidney 293T cells (American Type Culture Collection (ATCC) CRL-3275) provided by Marc Diamond were maintained in DMEM supplemented with 10% FBS. H4 neuroglioma cells (HTB-148) where purchased from ATCC and were maintained in DMEM supplemented with 10% FBS. H4 cells were stably transduced with a lentivirus encoding a FRET-based tau probe comprising the 344 to 378 residues of human P301L mutant tau fused to mTurquoise2, a self-cleaving 2A peptide, and 344 to 378 of human P301L mutant tau fused to mNeonGreen (53). All cells were cultured with 1× penicillin–streptomycin (Corning 30–002-CI) and maintained at 37 °C and 5% CO2 in a humidified atmosphere.

### Proteins, antibodies, and plasmids

RAP was expressed in *Escherichia coli* (54). Full-length tau (2N4R; SP-495) and tau MBD (SP-496, Leu243 - Glu372) were purchased from R&D Systems. His-tagged recombinant human tau variants 2N4R, 0N3R, 2N3R, and mutated tau proteins were expressed in *E. coli* and purified. His-tagged phosphorylated 2N4R tau was produced in SF9 cells. Cells were not treated with phosphatase inhibitor during production, resulting in an intermediate tau phosphorylation state (34). SF9 cells of 10 × 175-cm^2^ with 80 to 90% confluency were infected with P3 or P4 recombinant baculovirus (multiplicity of infection 5–10) and incubated at 27 °C for 48 to 72 h. Cells were pelleted at 500*g* for 5 min and resuspended in 30 ml lysis buffer containing 50 mM Tris–HCl, 100 mM NaCl, 10% glycerol, 5 mM imidazole, 0.5 mM tris(2-carboxyethyl)phosphate, 0.1 mM PMSF, benzonase 30 U/ml, and 1× Halt protease, and phosphatase inhibitor cocktail (Thermo Fisher Scientific 1861282). Cells were crushed in a French press twice, suspension was boiled for 20 min in a 100 °C water bath, cooled down on ice for 15 min, and centrifuged at 15,000*g* for 30 min to remove debris. Lysate was run on a HisTrap affinity column, and collected fractions were dialyzed into PBS containing 0.5 mM tris(2-carboxyethyl)phosphate. His tag was removed by enzymatic cleavage. Subsequently, 2N4R tau harboring the 6A or 6E mutations was generated by converting T181, S199, S202, S396, S400, and S404 to alanine (6A mutant) or glutamic acid (6E mutant). 3XKQ and 9XKQ 2N4R tau were generated in *E. coli* by mutating lysine residues 311, 317, and 321 or lysine residues 311, 217, 321, 340, 343, 347, 353, 369, and 375 to glutamine residues. Mouse anti-SORL1 antibody was purchased from BD Biosciences (BD Transduction Laboratories, 611860, used for Western blotting). Rabbit anti-SORL1 antibody (D8D4G) was purchased from Cell Signaling Technology (79322, used for immunohistochemistry). Chicken anti-GAPDH was obtained from Abcam (ab83956). SORL1 plasmid was provided by Claus Petersen, Aarhus University, Sweden (pcDNA3.1/zeo SORL1 Fl) (19). SORL1 G511R and N1358S plasmids were prepared by mutating the WT SORL1 plasmid, mutants were generated by VectorBuilder. Recombinant Human SORL1 VPS10 domain containing aa 82 to 753 was purchased from R&D Systems (9880-LA). Recombinant full-length human SORL1 (FL SORL1) his-tag protein was purchased from R&D Systems (11083-LA). The rabbit anti-LRP1 polyclonal (R2629) antibody was used to inhibit ligand binding to LRP1 as previously described (55).

### Tau internalization assays

Cellular internalization assays were conducted as previously described (11, 37, 56). Twelve-well culture dishes were seeded with CHO (2 × 10^5^ cells per well), HEK293T FRET reporter (1 × 10^5^ cells per well) cells, or H4 cells (0.5 × 10^5^ cells per well). Cells were cultured overnight in DMEM (H4 and HEK293T) or DMEM/F12 (CHO) with 10% FBS without antibiotic. The following day, cells were transfected with plasmids containing SORL1 or siRNA to knockdown SORL1 as described below. After transfection or knockdown, cells were incubated in assay media (DMEM supplemented with 1.5% bovine serum albumin and 20 mM Hepes) for 1 h and then incubated with assay media containing 20 nM ^125^I-labeled tau (2N4R; R&D Systems, Inc; SP-495) in the presence or absence of 1 μM RAP for specified times. In some experiments LRP1 was inhibited by coincubation with 300 μg/ml R2629 anti-LRP1 antibody. Following incubations, cells were washed 3× in Dulbecco's phosphate-buffered saline (DPBS), and then incubated with 0.5 ml of trypsin/proteinase K (to dissociated surface associated proteins), and then pelleted by centrifugation. The amount of internalized ^125^I-labeled tau was determined my measuring the radioactivity associated with the pellet using a gamma-counter.

### Transfections

Twenty-four hours after plating, cells were transfected with pcDNA3.1/zeo plasmid containing the SORL1 gene, or SORL1 harboring the G511R or N1358S mutant using 0.75 μg DNA per well *via* PEI transfection reagent at a ratio of 6 μl PEI:1 μg DNA. Transfection with empty vector was used as control. Cells were incubated with transfection reagent for 10 h during the day, then media were replaced with antibiotic-free media supplemented with 10% FBS and incubated overnight. Twenty-four hours after transfection, the tau internalization assay was performed as described previously. Transfection efficiency was confirmed *via* Western blot.

### siRNA knockdown

For tau uptake assays, H4 cells were plated at 0.5 × 10^5^ cells/well on a 12 well culture dish in DMEM + 10% FBS without antibiotic. The following day, cells were incubated with 25 nM ON-TARGETplus siRNA SMARTpool human SORL1 (Horizon L-004722-00-0005) or ON-TARGETplus Non-targeting Control Pool (Horizon D-001810-10-05) using 2 μl per well of DharmaFECT 1 Transfection Reagent (Horizon T-2001). Cells were incubated with siRNA for 48 h, and then media were replaced with assay media for tau uptake assays. Knockdown efficiency was confirmed *via* Western blot. For tau seeding assay in H4 FRET reporter line, cells were plated at 10^4^ cells/well in a 96 well plate. The next day, cells were transfected with 25 nM siRNA using 0.5 μl Lipofectamine 3000 Transfection Reagent (Invitrogen L3000015) per well in culture medium (in DMEM supplemented with 10% FBS). Tau seeds were added 24 h after transfection and incubated for 48 h.

### Surface plasmon resonance

Binding of tau isoforms 2N4R, 2N3R, and 2N4R tau harboring the 6A, and 6E mutations, hyperphosphorylated 2N4R tau produced in Sf9 cells, 3XKQ, and 9XKQ tau to FL SORL1 or SORL1 VPS10 were assessed using a Biacore 3000 optical biosensor system (GE HealthCare Life Sciences) or a Biacore 8K (Cytiva) essentially as described (11, 57). Recombinantly produced FL SORL1 or SORL1 VPS10 were immobilized *via* amine coupling to a CM5 Biacore sensor chip, and single cycle titrations were performed by serial injections from low to high concentration (3.8, 11.5, 34.4, 103.3, and 310 nM) with a 3.5-min injection time. Unless otherwise noted, all experiments were conducted in 0.15 M NaCl, 0.05% Tween 20, and 1 mM CaCl_2_ pH 7.4. Between sample runs, sensor chip surfaces were regenerated with 15-s injections of 100 mM phosphoric acid (pH ∼2.5) at a flow rate of 100 μl/min. For experiments to determine if VPS10 can compete binding of tau to SORL1 or VPS10, receptors were immobilized as above, and then the “A-B-A” method was used assay binding inhibition. Subsequently, 25 nM tau was flowed over each receptor in the presence or absence of 500 nM VPS10. For pH experiments, LRP1 or SORL1 were immobilized by amine coupling to a CM5 sensor chip, and the binding of tau was assayed as above in 0.01 M Hepes-buffered saline, 0.15 M NaCl, 0.05% Tween 20, 1 mM CaCl_2_ pH 7.4 or 0.01 M Mes, 0.15 M NaCl, 0.005% Tween 20, and 1 mM CaCl_2_ pH 5.5 buffer.

### Kinetic analysis of SPR data

Single cycle titration data were analyzed by fitting the titration to a 1:1 interaction model (58) or by an equilibrium fit of the individual data to a pseudo-first-order process to obtain values of Req for each concentration, then the Req values were plotted as a function of total concentration of tau. These equilibrium data were fit to a binding model using nonlinear regression analysis available in GraphPad vs 9 (https://www.graphpad.com).

### Microscopy

H4 cells were grown on 18-chamber microscope slides in DMEM supplemented with 10% FBS until subconfluent. In some cases, cells were transfected with GFP-tagged SORL1 as described, and tau incubation was performed 48 h posttransfection. Cells were serum starved by incubating in DMEM in the absence of FBS for 1 h prior to the experiment. Recombinant 2N4R tau (R&D SP495) was labeled using Alexa Flour 594 protein labeling kit according to manufacturer’s instructions (Life Technologies). Cells were incubated with 40 nM tau conjugated to Alexa Flour 594 in DMEM at 37 °C for 2 h. Cells were washed with DPBS to remove unbound tau and the fixed in 4% paraformaldehyde for 10 min at room temperature. Slides were washed with DPBS, and in some cases anti-SORL1 antibody (Cell Signaling SORL1 (D8D4G) Rabbit mAb 79322) was used to label endogenously expressed SORL1. VECTASHIELD Antifade with 4′,6-diamidino-2-phenylindole (DAPI, Vector laboratories H-1200) mounting media was used to stain the nuclei. Fluorescent images were acquired using a CSU-W1 spinning disk confocal system (Nikon) in the Center for Innovative Biomedical Resources Confocal Microscopy Facility at the University of Maryland School of Medicine. Images were acquired with a 60 × 1.49 numerical aperture oil-immersion objective as z-stacks with a step size of 0.1 μm. Imaris Bitplane 10.0 was used to generate images for publication and analyze for regions of colocalization. Colocalization analysis was performed in the Coloc module of Imaris Bitplane by masking data to analyze a single cell, thresholding to distinguish signal from noise, and determining the % of Material A (tau) that colocalized with Material B (SORL1).

### Tau seeding FRET biosensor assay

Human brain homogenates were prepared from an AD Braak VI brain and one healthy control brain from the Massachusetts Alzheimer's Disease Research Center Brain Bank. Briefly, 100 mg of frontal cortex tissue (Brodmann area 8/9) was thawed and homogenized in 500 μl of PBS with protease inhibitor (Roche) by 30 up and down strokes in a glass Dounce homogenizer. The homogenate was centrifuged at 10,000*g* for 10 min at 4 °C. The supernatant was aliquoted, and a bicinchoninic acid assay (Thermo Fisher Scientific Pierce) was performed according to manufacturer's instructions to quantify total protein concentration. Soluble HMW-SEC tau was isolated from homogenate using SEC on a Superdex200 10/300 Gl column (#17–5175–01; GE HealthCare) as described previously (8). Total tau concentration was measured by Western blot using a rabbit polyclonal anti-human tau antibody (A0024, Dako) and serial dilutions of recombinant tau 441 (Sigma-Aldrich, T0576). The Tau RD P301S FRET Biosensor 293T cells (ATCC CRL-3275) were reverse transfected in Costar Black (Corning) clear bottom 96-well plates, using 0.3 μl/well *trans*-IT X2 reagent (MiRus) in 10 μl/well Opti-MEM according to manufacturer’s protocol with 100 ng/well empty pcDNA3 plasmid (mock condition) or pcDNA3 plasmid encoding for the WT, G511R, N1358S SORL1 proteins. A total of 6 × 10^5^ cells/well were seeded in a final volume of 100 μl. After 24 h, transfection medium was removed, cells were washed with sterile PBS, and incubated with 300 ng/well tau diluted in Opti-MEM (final volume 50 μl). The next day, each condition was tested at least in quadruplicate. The following day, cells were collected using trypsin and transferred into 96-well U-bottom plates (Corning) using 10% FBS culture media to neutralize trypsin. Cells were pelleted at 1200*g* for 10 min, resuspended in cold 2% paraformaldehyde for 10 min, pelleted at 1200*g*, and resuspended in 200 μl of PBS. Samples were run on the MACSQuant VYB (Miltenyi) flow cytometer for the quantification of cyan flourescent protein and FRET. Tau seeding was quantified by multiplying the percent of FRET-positive cells by the median fluorescence intensity of those cells, as described previously (59). About 40,000 cells per well were analyzed. Data were analyzed using FlowJo (https://www.flowjo.com) software (BD Biosciences). For the seeding assay performed in stable H4 reporter line, 15′000 cells/well were plated.

### SDS-PAGE and Western blot

Cell cultures were collected in radioimmunoprecipitation assay lysis buffer and analyzed by Western blotting as previously described (11). Equal amounts of protein from each sample were mixed with loading buffer with or without 100 mM DTT, boiled for 5 min, resolved by electrophoresis on a Novex 4 to 12% Tris-Glycine Mini Protein Gel, and transferred to polyvinylidene difluoride membranes for Western blot analysis. Membranes were blocked with Odyssey blocking buffer and incubated with anti-SORL1 antibody at a concentration of 1:1000 or anti-GAPDH 1:1000 overnight at 4 °C. The membrane was washed three times with 0.05% Tween-20 in Tris-buffered saline, and the antibody binding to membrane was detected with IRDye 680RD or 800 anti-mouse, anti-rabbit or anti-chicken IgG secondary antibody (LI-COR Biosciences) at a concentration of 1:10,000. The membrane was then washed three times with 0.05% Tween-20 in Tris-buffered saline and imaged using a LI-COR Odyssey Infrared Imaging System.

### Experimental design and statistical analysis

All results are represented as mean ± SEM or SD, as indicated. Data were analyzed for significance using one-way ANOVA, or two-way ANOVA, with Tukey's multiple comparisons post tests, as indicated using GraphPad vs 9 software. A *p* value of <0.05 was set as the threshold for significance.

## Data availability

All of the data are contained within the manuscript.

## Supporting information

This article contains supporting information.

## Conflict of interest

The authors declare that they have no conflicts of interest with the contents of this article.
