# Supplementary figures and images for: SORL1 is a receptor for tau that promotes tau seeding
Source: J Biol Chem. 2024 Apr 23;300(6):107313. doi: 10.1016/j.jbc.2024.107313 (PMC11145553; doi:10.1016/j.jbc.2024.107313)

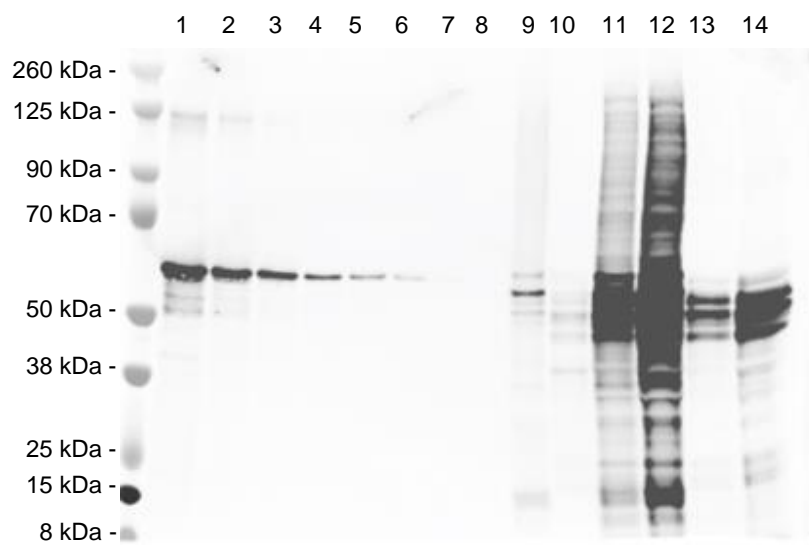

Supplement: Supporting Figure S1 [file mmc1.pdf]
