# Supplementary material for: SORL1 is a receptor for tau that promotes tau seeding
Source: J Biol Chem. 2024 Apr 23;300(6):107313. doi: 10.1016/j.jbc.2024.107313 (PMC11145553; doi:10.1016/j.jbc.2024.107313)
Supplement: Supporting information [file mmc2.docx]

**Supplemental Figure 1. Immunoblot of brain lysates and SEC fractions of AD and CT patient brain samples.** Noted amounts of recombinant 2N4R tau (lanes 1-8: 1, 300 ng; 2, 150 ng; 3, 75ng; 4, 37.5 ng; 5, 18 ng; 6, 9 ng; 7, 5 ng; 8, 3.5 ng ); HMW (lane 9, 3.5 μg) and LMW SEC (lane 10, 35 μg) fractions from an AD patient brain; and brain lysate from the same AD (lane 11, 3.5 μg; lane 12, 10 μg) or age mached control lysates (lanes 13, 3.5 μg, lane 14, 10 μg). Samples were separated on a 4-12% Bis-Tris gel via electrophoresis and immunoblotted using rabbit anti-tau antibody (Dako, A0024).
